# Microscopic and Molecular Evidence of the First Elasmobranch Adomavirus, the Cause of Skin Disease in a Giant Guitarfish, Rhynchobatus djiddensis

**DOI:** 10.1128/mBio.00185-18

**Published:** 2018-05-15

**Authors:** Jennifer A. Dill, Alvin C. Camus, John H. Leary, Terry Fei Fan Ng

**Affiliations:** a Department of Pathology, College of Veterinary Medicine, University of Georgia, Athens, Georgia, USA; NIH; Virginia Polytechnic Institute and State University

**Keywords:** cartilaginous fish, giant guitarfish, adomavirus, metagenomics, pathology, skin disease, virus discovery

## Abstract

Only eight families of double-stranded DNA (dsDNA) viruses are known to infect vertebrate animals. During an investigation of papillomatous skin disease in an elasmobranch species, the giant guitarfish (Rhynchobatus djiddensis), a novel virus, distinct from all known viral families in regard to particle size, morphology, genome organization, and helicase phylogeny was discovered. Large inclusion bodies containing 75-nm icosahedral viral particles were present within epithelial cell nuclei in the proliferative skin lesions. Deep metagenomic sequencing revealed a 22-kb circular dsDNA viral genome, tentatively named guitarfish “adomavirus” (GAdoV), with only distant homology to two other fish viruses, Japanese eel endothelial cell-infecting virus (JEECV) and a recently reported marbled eel virus. Phylogenetic analysis of the helicase domain places the guitarfish virus in a novel clade that is equidistant between members of the *Papillomaviridae* and *Polyomaviridae* families. Specific PCR, quantitative PCR, and *in situ* hybridization were used to detect, quantify, and confirm that GAdoV DNA was localized to affected epithelial cell nuclei. Changes in the viral titer, as well as the presence of a hybridization signal, coincided with the progression and then final resolution of gross and microscopic lesions. The results indicate that GAdoV is the causative agent of the proliferative skin lesions.

## IMPORTANCE

Cartilaginous fish, including the sharks and rays, evolved from ancestral fish species at least 400 million years ago. Even though they are the descendants of one of the most ancient vertebrate lineages, reports of viral diseases in these species are rare and poorly documented. Deep sequencing revealed a highly divergent virus, tentatively named guitarfish adomavirus, that is distantly related to known papillomaviruses and polyomaviruses. Out of the eight predicted viral genes, only the helicase could be identified as viral by sequence homology searches (BLAST), exemplifying the difficulties of discovering novel viruses within seas of unidentifiable “dark matter” associated with deep sequencing data. The novel adomavirus represents the first viral genome shown to cause clinical disease in a cartilaginous fish species, the giant guitarfish. Our findings demonstrate that emerging fish viruses are fertile ground to expand our understanding of viral evolution in vertebrates.

## OBSERVATION

Viruses do not leave a fossil record. Their evolutionary history is usually deduced from the molecular phylogeny of viruses found in extant hosts, except in occasional specimens where viruses are exceptionally well preserved by extremely cold or stable environments (1–4). Ancestral fish emerged approximately 530 million years ago (MYA) during the Cambrian explosion. Comparative genomic studies of new viruses from extant fish to other well-characterized vertebrate viruses provide an important key to further our understanding of long-term viral evolution (5, 6).

Currently, there are eight families of double-stranded DNA (dsDNA) viruses known to infect vertebrates, including the *Polyomaviridae*, *Papillomaviridae*, *Adenoviridae*, *Herpesviridae*, and *Alloherpesviridae*, and three families in the proposed supergroup Megavirales, namely, *Poxviridae*, *Asfarviridae*, and *Iridoviridae*. The origins and ancient evolutionary history of these families remain elusive (7–10). Infections by polyomaviruses and papillomaviruses in fish have been described only recently (6, 11–13). In addition, two unclassified fish viruses distantly related to the *Polyomaviridae* have been reported. The first, Japanese eel (Anguilla japonica) endothelial cell-infecting virus (JEECV) is the cause of viral endothelial cell necrosis of eels (VECNE) disease (14, 15). The second example of this emerging clade was isolated from diseased marbled eels (Anguilla marmorata) in Taiwan (16). Although both viruses encode a large T antigen typical of polyomaviruses found in bony fish, the remainder of their ~15-kb circular dsDNA genomes showed little similarity to other known viral sequences.

Elasmobranch fishes, including the sharks, rays, guitarfishes, and others, evolved from ancestral species at least 419 MYA during the early Devonian period. Compared to bony teleost fish, such as eels, examples of viral diseases in elasmobranchs are rare (17, 18). During an investigation into the etiology of a proliferative skin disease in a giant guitarfish (17), two viral genomes were identified. One, giant guitarfish polyomavirus 1, was the first viral genome described from an elasmobranch species and represents the smallest known polyomavirus genome (11). Here, we present a description of the second viral genome, highlighting its similarities to JEECV and the marbled eel virus, as well as histopathology, quantitative PCR, and *in situ* hybridization findings that establish it as the etiologic agent of a proliferative skin disease in the giant guitarfish. A concurrent study focused on viral gene evolution and classification (N. L. Welch et al., submitted for publication) suggests that the helicase and capsid genes of the guitarfish virus, JEECV, and marbled eel virus share a complex evolutionary history with polyomaviruses, papillomaviruses, and adenoviruses and that these three fish viruses may represent a novel viral lineage—adomavirus. Here, we aim to document the first description of disease caused by this new clade of adomavirus in an elasmobranch, the giant guitarfish.

### Disease presentation.

**FIG 1:**
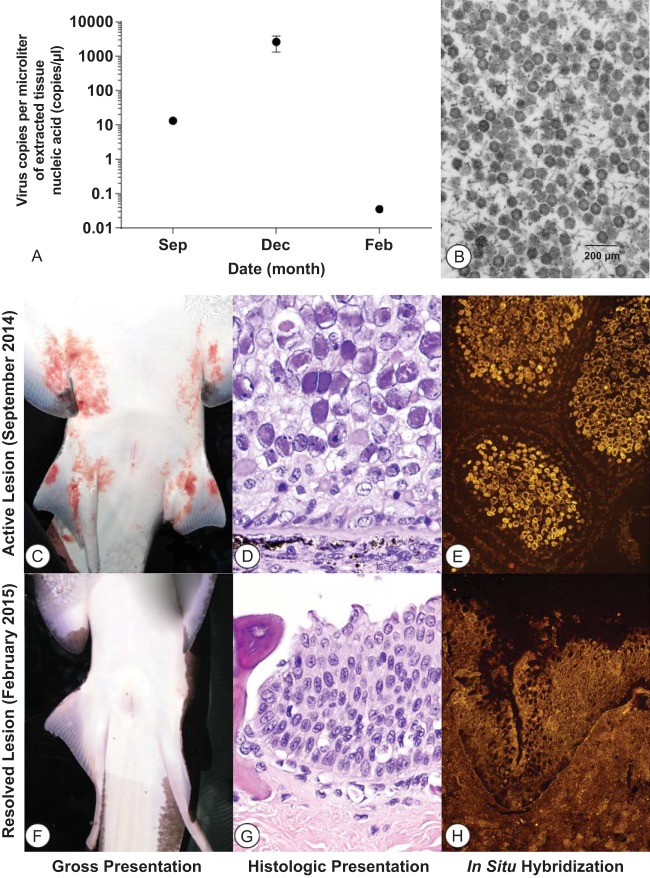
Progression and regression of adomavirus-induced skin lesions in a giant guitarfish over an 18-week period. (A) Quantification of virus using an adomavirus-specific qPCR. (B) Transmission electron microscopic image of an epithelial cell nucleus with a large array of nonencapsulated, 75-nm, icosahedral, viral particles during active infection in September. (C to H) The images in panels C to H compare lesion changes and FISH results. (C) Gross image of guitarfish with active lesions in September. The ventral skin has multiple reddened, raised areas of epithelial proliferation. (D) Histologically, the diseased skin had numerous epithelial cell nuclei enlarged by amphophilic viral inclusions that marginated nuclear chromatin. (E) Using FISH, strongly positive fluorescent signals were localized to affected epithelial cell nuclei with an adomavirus probe. (F) Gross image of the guitarfish demonstrating normal skin 18 weeks later in February. (G and H) Epithelial cells and their nuclei were microscopically normal, with no viral inclusions (G), and no hybridization signal was detected using FISH (H).

Grossly, pink to red, variably sized, villus-like skin lesions were widely distributed over ventral surfaces, involving the perioral area, claspers, pectoral and pelvic fins and their bases (Fig. 1C). A heavily melanized lesion was present on pigmented areas of the caudal fin. Microscopic examination revealed papillary proliferation of the epidermis, with approximately 75% of nuclei containing hyaline, amphophilic inclusions that marginated nuclear chromatin (Fig. 1E). Electron microscopy (17) revealed large arrays of icosahedral, ~75-nm virions within affected epithelial cell nuclei (Fig. 1B). Presented in greater detail in a previous case report (17), these findings prompted an investigation using deep metagenomic sequencing that demonstrated the novel adomaviral genome.

### A novel adomavirus coinfecting the epithelial cell lesion with a polyomavirus.

**FIG 2:**
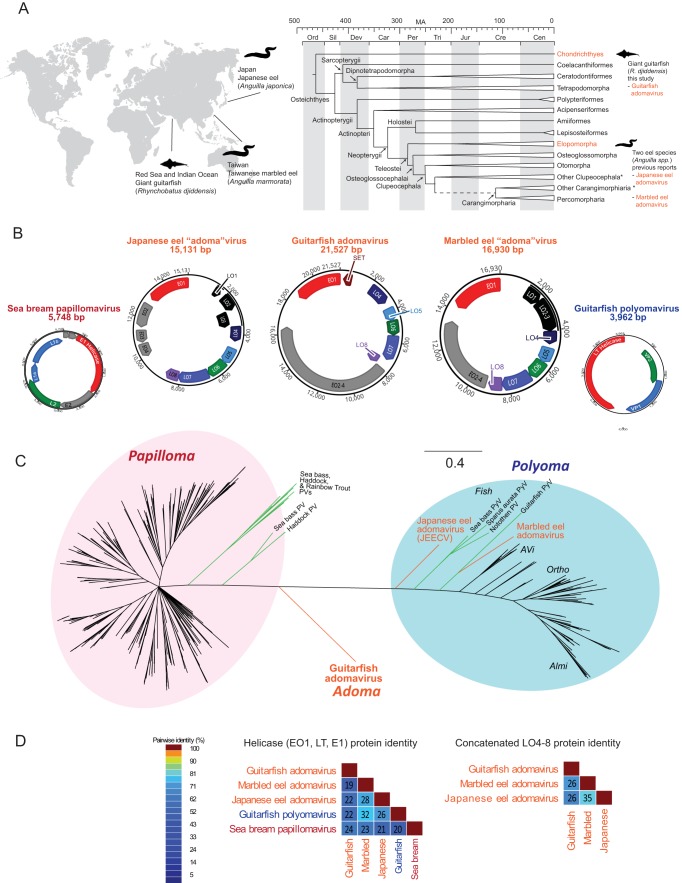
Host range, phylogeny, and genomic characteristics of guitarfish adomavirus. (A) Spatial range, host phylogeny, and evolution time scale of the giant guitarfish host species from which adomaviruses have been characterized. The host phylogeny was reconstructed according to a previous comprehensive study (24). The fish host clade detected with adomavirus infection is labeled red. For clarity, extensive diversification of Clupeocephala and Carangimorpharia during the last 250 million years is condensed. (B) Genome size and organization of guitarfish adomavirus in comparison to sea bass papillomavirus and guitarfish polyomavirus. The viral genome from marbled eel and Japanese eel (KU221231 and NC_015123) are tentatively renamed “adomavirus” in reference to the classification analysis by Welch et al. (submitted). All adomavirus genomes, as shown in panel C, are larger than 15 kb. (C) Bayesian inference phylogeny of the helicase domain. The three adomaviruses from guitarfish, Japanese eel, and marble eel are shown in orange. Fish papillomaviruses (PV) and polyomaviruses (PyV) are shown with green branches. Even though the Japanese eel and marbled eel viruses contain a helicase that is homologous to polyomavirus LT, further comparative genomics (Welch et al., submitted) indicated that the eel helicase phylogeny is the result of chimerization, and the two eel viruses are *de facto* adoma-like viruses as recognized by their genome size, organization, and virion structure. PV, papillomavirus; PyV, polyomavirus. (D) Pairwise protein identity comparisons between representative adomaviruses, polyomavirus, and papillomavirus.

Viral metagenomics performed directly on biopsied lesion tissue identified giant guitarfish polyomavirus 1 (GfPyV1) (11). However, the 75-nm virus particles seen with electron microscopy were not consistent with the 45- to 60-nm size range typical of polyomaviruses. Subsequent next-generation sequencing (NGS) analysis revealed a divergent viral helicase sequence. Based on this, *de novo* assembly and alignments were used to construct the complete circular 22-kb genome of a second virus, guitarfish adomavirus (GAdoV) (Fig. 2B), that was much larger than the 4-kb genome of GfPyV1. In the final assembly, GAdoV was confirmed by 779,443 reads and >4,100× average coverage across the genome (see Fig. S1 in the supplemental material). The GC content of the virus was 42%. In retrospect, despite the abundance of adomavirus reads in the NGS data, the virus was initially difficult to detect, because with the exception of the conserved helicase domain, the majority of the genome was unidentifiable using BLAST searches for viral sequences.

10.1128/mBio.00185-18.1 FIG S1  Coverage analysis of the next-generation sequencing of guitarfish adomavirus. Download FIG S1, PDF file, 1 MB. Copyright © 2018 Dill et al. 2018 Dill et al. This content is distributed under the terms of the Creative Commons Attribution 4.0 International license.

The genome organization and viral proteins of GAdoV were highly divergent compared to known viral families (Fig. 2). This adomavirus genome is circular and encodes two cassettes of bidirectionally transcribed genes with eight open reading frames (ORFs), namely, EO1, EO2-4, LO4, LO5, LO6, LO7, and LO8, as well as a SET [Su(var)3-9 Enhancer-of-Zeste and Trithorax] homolog (Fig. 2B). The shortest ORF, SET, encodes 176 amino acids (aa), and the longest ORF, the EO2-4 gene, encodes 2,889 aa. The length of the EO2-4 gene alone is larger than the typical whole genomes of papillomaviruses and polyomaviruses.

The most conserved ORF among the guitarfish and eel viruses is the EO1 helicase. The predicted EO1 protein shows a segment with distant similarity to the superfamily 3 helicase domains of both the E1 proteins of papillomaviruses (<27% protein identity) and the LT proteins of polyomaviruses (<23% protein identity). Phylogenetic analysis of the helicase domain in EO1 showed that GAdoV constitutes a novel clade, with equal distance to members of the viral families, *Papillomaviridae* and *Polyomaviridae* (Fig. 2C). The two previously described eel viruses cluster with the polyomaviruses, even though their genome sizes and organization are clearly different from them (Fig. 2B). This perhaps implies that the helicase/EO1 of the eel viruses may have evolved independently from the rest of the genome, possibly resulting from a recombination event between their viral ancestors. Concordantly, the DNAJ-like domain, a unique hallmark of polyomavirus LT proteins (19), was detected in the two eel viruses, but not in GAdoV. In the guitarfish virus, the EO2-4 gene encodes a >300-kDa protein with an N-terminal domain that shows protein identities (~35% by BLAST) to the catalytic subunit of archaeal-eukaryotic DNA primases (AEPs). The three adomaviruses share a 7-kb tandem array of homologous LO4 to LO8 ORFs that share 26 to 35% protein identities (Fig. 2D) and do not resemble any papillomavirus or polyomavirus proteins by sequence homology or length. However, a structural homology search using HHpred showed that they share weak homology (score, 53; identities, 18%) to adenoviruses, which also encode a tandem array of multiple capsid subunits.

Molecular findings suggest that only infection by GAdoV, not the polyomavirus, was productive in the guitarfish. Metagenomics with random amplification was performed to achieve agnostic NGS sequencing, revealing a much larger number of reads in affected tissue for the adomavirus (779,443) compared with the polyomavirus (634). Even though comparison of the number of NGS reads is only semiquantitative, a substantially larger amount of adomavirus DNA was present for sequencing than that of the polyomavirus. Both conventional and real-time PCR amplicons were reconfirmed by Sanger sequencing for the presence of the adomaviral nucleic acids. Furthermore, as detailed below, subsequent fluorescent *in situ* hybridization (FISH), using separate adomavirus-specific and polyomavirus-specific probes, demonstrated only the presence of adomavirus nucleic acid within histological lesions.

### Adomaviral infection as the cause of guitarfish skin lesions.

To investigate disease progression, quantitative PCR (qPCR), histopathology, and FISH were performed at three time points at 8- to 10-week intervals (Fig. 1) following the rapid appearance of erythematous raised skin lesions in September 2014 (Fig. 1C). The lesions remained grossly visible but had begun to regress in December and had completely resolved by February 2015. Tissue samples collected in September and December revealed villus-like proliferation of the epidermis, accompanied by widespread karyomegaly and large numbers of amphophilic intranuclear inclusion bodies (Fig. 1E). GAdoV DNA levels remained high during this time, with qPCR levels increasing from 1.21 × 10^5^ ± 2.53 × 10^3^ copies/µl to 2.42 × 10^7^ ± 1.2 × 10^7^ copies/µl during the approximately 10 weeks while lesions were active (Fig. 1A). Additionally, strong positive hybridization signals localized GAdoV DNA to the intranuclear inclusions of affected epithelial cells (Fig. 1G). Hybridization signals were not observed in unaffected basal cells of the epithelium or in adjacent dermal connective tissues. Using the GfPyV1-specific probe, no hybridization signal to the polyomavirus was detected in any cell type within the tissue sections, suggesting that the level of GfPyV1 was lower than the limit of detection for the FISH technique.

By February 2015, skin lesions in the guitarfish were no longer grossly visible (Fig. 1D). A follow-up biopsy sample revealed an epithelium of typical height and cellular morphology, devoid of the previously observed papillary proliferations, karyomegaly, and nuclear inclusions (Fig. 1F). While GAdoV DNA remained quantifiable by qPCR, it had decreased to an extremely low level of 3.26 × 10^2^ ± 4.26 × 10^1^ copies/µl (Fig. 1A) and FISH signals were no longer detected (Fig. 1H). In all samples, polyomavirus (GfPyV1) DNA was not detected by FISH. Taken collectively, the results of metagenomic sequencing, the rise and fall of adomavirus DNA levels, and the presence and disappearance of adomavirus hybridization signals that occurred over time in conjunction with the development and resolution of gross and microscopic lesions support the conclusion that GAdoV is the etiologic agent of skin lesions in the guitarfish.

Exploration of the virosphere has been greatly enhanced by the use of metagenomic and high-throughput sequencing approaches. Many aspects of viral origins and evolution remain a mystery, but as descendants of the earliest vertebrates, dating back at least 500 million years, contemporary elasmobranch and teleost fishes may be central to our understanding of virus evolution in early chordates (Fig. 2A). Due to their ancient origins, it is not surprising that viruses infecting these species are divergent from better-characterized mammalian and avian counterparts. It is also possible to imagine that evolutionary bottlenecking events during the transition to life on dry land could have resulted in the loss of some groups of viruses in terrestrial vertebrates. As a result, fish are likely to represent a rich source of viruses that can help define our understanding of virus evolution and enrich our knowledge of phylogenetic relationships between viral families (5, 6).

In this article, we report a novel viral genome (GenBank accession number MF946548), tentatively named guitarfish adomavirus, and show molecular evidence of its pathogenicity. This virus and a previously described polyomavirus, GfPyV1, were discovered coinfecting proliferative skin lesions (17) in a giant guitarfish. Guitarfish adomavirus represents the first viral genome and infection to which clinical disease has been attributed in an elasmobranch. Results of qPCR and FISH studies, in conjunction with histopathologic and electron microscopic findings, strongly support the previous association of this unusual viral pathogen as the cause of skin lesions in this guitarfish. Furthermore, sequence-independent metagenomics, as well as additional protein and structural analyses (Welch et al., submitted), indicate that this genome may be a prototype member of a new group of viruses, collectively called adomaviruses.

The 75-nm icosahedral particles of GAdoV are similar in size and shape to the two other related viruses from the Japanese and marbled eel (14–16) (Fig. 2) and bear some resemblance to the 80- to 100-nm virions of adenoviruses. However, they are distinctly different from the knobby 45- to 60-nm virions of polyomaviruses and papillomaviruses or the 120-nm capsids of herpesviruses. The genome size of guitarfish adomavirus is also strikingly large compared to other vertebrate dsDNA viruses with a circular genome. The 22-kb genome of GAdoV is several times larger than the 7- to 8-kb genomes of papillomaviruses and 4- to 5-kb genomes of polyomaviruses found in birds and mammals. Although eight viral ORFs were identified, only one, the nonstructural helicase protein, had enough sequence homology to other viruses to enable our initial discovery. Detection of the viral helicase made it possible to identify and assemble the remaining over half a million “dark matter” reads (20), most of which are now predicted to be highly divergent structural proteins (LO4 to LO8). This highlights how a highly divergent virus can be unrecognizable even when NGS is performed. However, characterization of GAdoV will provide a sequence database baseline to facilitate the discovery of related sequence homologies and additional adomaviruses in the future. Additional experiments (Welch et al., submitted) could further investigate the weak structural homology between the adomavirus LO4 to LO8 proteins and adenovirus capsid proteins.

Polyomaviruses and other unique DNA viruses have been described only recently in fish, and little is known regarding their host range or disease potential. Now, in addition to the Japanese and marbled eel viruses (14–16), discovery of the guitarfish adomavirus in an elasmobranch has expanded the host range to an extant descendant of one of the earliest fish lineages (Fig. 2A). The existence of an adomavirus in a cartilaginous fish leads us to hypothesize that such viruses may have existed in a common ancestral fish species prior to the diversification of the jawed fishes into cartilaginous and bony fish classes. As a result, the host range of adomaviruses is likely to encompass many additional fish groups.

Despite their similarities in size, ultrastructure, and genome organization, as well as their association with fish species, the diseases caused by the eel viruses are highly dissimilar from that produced by GAdoV. The Japanese eel virus produces epizootics of acute fatal disease characterized by systemic hemorrhage and tissue necrosis resulting from endothelial cell infection and damage (14, 15). In contrast, GAdoV caused a chronic self-limiting infection of the skin that resulted in tumor-like epithelial cell proliferation in an individual guitarfish. Additional cases did not occur among conspecifics in the same tank, which suggests that the virus is not highly transmissible or does not always cause overt clinical signs.

As evidenced by results of qPCR and FISH studies, in conjunction with histopathologic and electron microscopic findings, we infer that GAdoV is the cause of the proliferative skin lesions in this guitarfish. These findings warrant further investigation of adomaviruses as emerging infectious agents capable of producing varied disease signs and lesions in different fish species. Additionally, this is the first report to describe coinfection by a polyomavirus and adomavirus in skin lesions of a single host. Although they represent only distantly related viral lineages, coinfection by the two viruses could provide fertile ground for horizontal recombination events to occur, which could be a driving force for viral evolution.

### Methods. (i) Sample collection and electron microscopy.

A juvenile male giant guitarfish Rhynchobatus djiddensis caught in the wild and measuring 148 cm and 13.5 kg was transported internationally to a public aquarium. Soon after arrival, the animal developed erythematous skin lesions on its ventral skin and tail that became raised and papillary over an 8-week period. Biopsy samples of the lesion were collected, fixed in 10% neutral buffered formalin, and submitted to the Department of Pathology, University of Georgia College of Veterinary Medicine for routine processing and histological evaluation. Additional samples were frozen at −80°C, and samples were fixed in a solution of 2% glutaraldehyde, 2% paraformaldehyde, and 0.2% picric acid in 0.1 M cacodylate-HCl buffer (pH 7.0 to 7.3) and processed for transmission electron microscopy (17). Cell culture was not attempted due to a lack of appropriate cell lines. Lesions resolved spontaneously over the 18 weeks that followed, during which time additional samples were reevaluated at 8- to 10-week intervals.

### (ii) Whole-genome sequencing using next-generation sequencing and bioinformatics.

Total DNA was extracted from skin lesions, and unbiased metagenomic sequencing was performed according to previously described protocols (2, 21). Briefly, tissues were homogenized and filtered to enrich for viral particles, the filtrate was depleted of host nucleic acid using nucleases, and unbiased sequence-independent amplification was performed using random priming. Specifically, nucleic acids from nuclease-resistant viral particles were extracted using the QIAquick viral RNA column purification system. Reverse transcription was performed using a 28-base oligonucleotide whose 3′ end consisted of eight random nucleotides (primer N1_8N, CCTTGAAGGCGGACTGTGAGNNNNNNNN). The second strand was synthesized using Klenow fragment DNA polymerase (New England BioLabs). The resulting double-stranded cDNA and DNA were then PCR amplified using AmpliTaq Gold DNA polymerase and a 20-base primer (primer N1, CCTTGAAGGCGGACTGTGAG). A duel-indexed sequencing library was prepared using the Nextera XT DNA sample prep kit (Illumina, San Diego, CA). After pooling, the final library was sequenced using the MiSeq sequencing system with 2 × 250-bp paired-end sequencing reagents (Illumina MiSeq Reagents V2; 500 cycles).

A total of 8,119.238 million reads were generated and analyzed as previously described (2). Adaptor and primer sequences were trimmed using VecScreen, while duplicate reads and low-sequencing-quality tails were removed using a Phred quality score of 10 as the threshold. The cleaned reads were *de novo* assembled using an in-house sequence assembler employing an ensemble strategy (2), and the resulting assembled contigs were compared to the GenBank protein database using BLASTx. Once the viral contigs were identified, a complete circular genome was constructed from the available contigs and reads using Geneious (Biomatters), which was also used to generate coverage analysis. The genomes of previously sequenced Japanese and marbled eel viruses (14, 16) were retrieved from GenBank using accession numbers NC_015123 and NC_030148.1

### (iii) Phylogenetic analysis.

The complete novel genome was analyzed together with the previously published Japanese eel and marbled eel virus genomes. Coding sequences of representative helicase genes were downloaded from GenBank. Helicase protein sequences were aligned using MAFFT with the E-INS-I alignment strategy and previously described parameters (3, 6). Bayesian inference trees were constructed using MrBayes, with every 50 generations of the Markov chain, Monte Carlo (MCMC) chain sampled, for a maximum of 1 million generations. The first 25% of MCMC samples were discarded as burn-in.

### (iv) Molecular screening of diseased guitarfish.

Guitarfish adomavirus-specific PCR screening and FISH probe primers were designed to target major genes (Table S1). Total nucleic acids were extracted from tissue using the QIAamp viral RNA minikit (Qiagen) that extracts both DNA and RNA. PCR was performed using One *Taq* DNA polymerase kits (New England BioLabs, Ipswich, MA) with a previously described touchdown thermocycling profile (21). The PCR protocol consisted of an initial denaturation step of 30 s at 94°C, followed by 45 cycles, with 1 cycle consisting of 30 s at 94°C and 30 s at 58°C with a −0.2°C touchdown each cycle, and 120 s at 68°C, followed by a final elongation step at 68°C for 5 min. PCR products were electrophoresed in a 1.5% agarose gel, and the resulting amplicons were verified by Sanger sequencing.

10.1128/mBio.00185-18.2 TABLE S1  Primers. Targeted genes, primer sequences, and product size for PCR of guitarfish adomavirus. Download TABLE S1, DOCX file, 0.01 MB. Copyright © 2018 Dill et al. 2018 Dill et al. This content is distributed under the terms of the Creative Commons Attribution 4.0 International license.

SYBR green-based quantitative PCR (qPCR) was used to assess the presence and quantity of viral DNA in skin lesions. Primers targeting the adomavirus LO7 gene (Table S1) were selected as the most promising. To obtain amplicon standards, the primer set was used in a standard PCR with DNA extracted from the guitarfish. The DNA was run on a 2% agarose gel, purified (Qiaquick gel extraction kit), and quantitated using a NanoDrop 2000 spectrophotometer (Thermo Fisher), and the concentration was adjusted to 1 ng/µl. Tenfold serial dilutions of this stock were made in water for qPCR standard curve generation. Preliminary analysis indicated that the 10^−1^ through 10^−8^ dilutions (10^−1^ to 10^−8^ ng) would cover the dynamic (linear) range of the assay (*R*^2^ ≥ 0.95). qPCRs for the tissue samples and standards were performed on a Bio-Rad IQ5 iCycler using iQ5 system software for analysis. One microliter of extracted DNA was added to each 25-µl reaction mix containing iQ SYBR green supermix (Bio-Rad) and 100 nmol each of the indicated primers. A two-step cycling program used an initial step of 3 min at 95°C, followed by 35 cycles, with 1 cycle consisting of 10 s at 95°C and 30 s at 60°C. Initial screening of all samples was done twice using one PCR well/sample. The final assessment of the presence of viral DNA was made on samples run in triplicate.

### (v) *In situ* hybridization of guitarfish tissue sections.

Fluorescent *in situ* hybridization (FISH) assays using a digoxigenin-labeled probe and a biotin-streptavidin method were adapted from previously described protocols (22, 23) using primers specific for guitarfish adomavirus (Table S1). Briefly, 3-µm sections of formalin-fixed paraffin-embedded skin were deparaffinized, rehydrated, and digested in ready-to-use proteinase K (Dako) for 15 min. The slides were placed in a Bio-Rad Frame-Seal incubation chamber and denatured with 100% formamide (Sigma) at 105°C for 5 min. The probe was diluted in hybridization solution, applied to slides, incubated at 105°C for 5 min, and then left to hybridize overnight in a 37°C humidified oven. On day 2, the slides were washed with 5× sodium chloride-sodium citrate buffer (1× SSC is 0.15 M NaCl plus 0.015 M sodium citrate) at room temperature followed by 2× SSC, then incubated at 37°C for up to 15 min. Sections were blocked with normal (healthy) goat serum (Rockland), conjugated with streptavidin-Alexa Fluor 532, and mounted with ProLong Gold with 4′,6′-diamidino-2-phenylindole (DAPI) (Molecular Probes). Negative controls were included. All steps were performed at room temperature unless otherwise noted. The slides were observed using an Olympus BX41 fluorescence microscope.

### Accession number(s).

The genome of the sequenced guitarfish virus was deposited in GenBank under accession number MF946548.
