# Supplementary figures and images for: Microscopic and Molecular Evidence of the First Elasmobranch Adomavirus, the Cause of Skin Disease in a Giant Guitarfish, Rhynchobatus djiddensis
Source: mBio. 2018 May 15;9(3):e00185-18. doi: 10.1128/mBio.00185-18 (PMC5954223; doi:10.1128/mBio.00185-18)

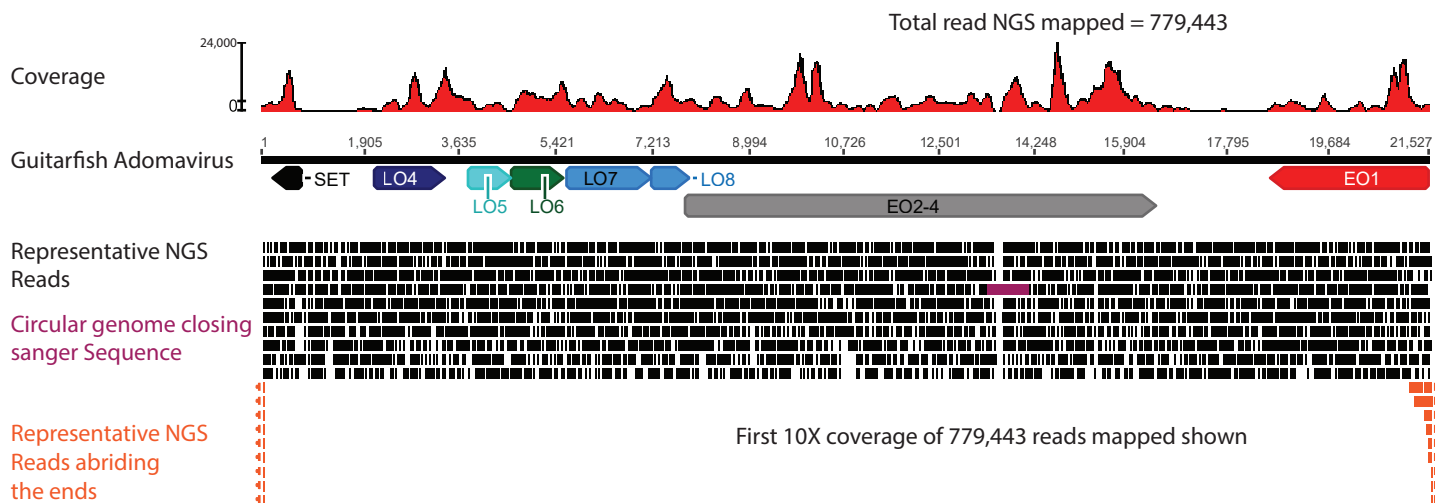

Supplement: FIG S1 [file mbo003183869sf1.pdf]
