# Supplementary material for: Microscopic and Molecular Evidence of the First Elasmobranch Adomavirus, the Cause of Skin Disease in a Giant Guitarfish, Rhynchobatus djiddensis
Source: mBio. 2018 May 15;9(3):e00185-18. doi: 10.1128/mBio.00185-18 (PMC5954223; doi:10.1128/mBio.00185-18)
Supplement: TABLE S1 [file mbo003183869st1.docx]

**Supplementary Table 1. Primers.** Targeted genes, primer sequences and product size for the PCR of guitarfish adomavirus.

| **Name** | **Sequence** | **Amplicon (bp)** | **Corresponding Gene** |
| --- | --- | --- | --- |
| AF | TCACTCACAGCTCCAAATGC | 390 | EO2-4 |
| AR | TCCGTACCTGCCACACACTA |  |  |
| BF | TGCTGTCAGAGGTGAAGGTG | 322 | EO1 (Helicase) |
| BR | ACCATTCCCCTTCCTAATGG |  |  |
| CF | CCAGAGGAAGATGGTGCAAT | 352 | LO7 |
| CR | CCTCCCTGGAATCGTCTGTA |  |  |
| CqPCRf | TGGGCCGTTCTATAACGAAA | 100 | LO7 |
| CqPCRr | AGCAAAGGAACTGCAGCCTA |  |  |
| DF | GGTACAGGCAGGACGACAAT | 340 | EO2-4 |
| DR | CTCGCTTATAATGCCGAAGC |  |  |
